# Prevalence and correlates of weight bias internalization in weight management: A multinational study

**DOI:** 10.1016/j.ssmph.2021.100755

**Published:** 2021-02-17

**Authors:** Rebecca L. Pearl, Rebecca M. Puhl, Leah M. Lessard, Mary S. Himmelstein, Gary D. Foster

**Affiliations:** a Center for Weight and Eating Disorders, Department of Psychiatry, Perelman School of Medicine at the University of Pennsylvania, Philadelphia, PA, USA; b Department of Clinical and Health Psychology, University of Florida, Gainesville, FL, USA; c Rudd Center for Food Policy and Obesity, University of Connecticut, Hartford, CT, USA; d Department of Human Development & Family Sciences, University of Connecticut, Storrs, CT, USA; e Department of Psychological Sciences, Kent State University, Kent, OH, USA; f WW International, Inc., New York, NY, USA

**Keywords:** Health behaviors, Internalized weight stigma, Obesity, Overweight, Psychosocial, Weight bias internalization, Weight management, Weight self-stigma

## Abstract

Weight bias internalization (WBI) is an understudied form of internalized stigma, particularly among treatment-seeking adults with overweight/obesity. The current study surveyed 13,996 adults currently engaged in weight management in the first multinational study of WBI. From May to July 2020, participants in six Western countries completed the Modified Weight Bias Internalization Scale (WBIS-M) and measures of weight change, health behaviors, psychosocial well-being, and health-related quality of life (HRQOL). Participants were majority white, female, middle-aged, and categorized as having overweight or obesity based on body mass index. Results showed higher mean WBIS-M scores among participants in the UK, Australia, and France than in Germany, the US, and Canada. Across all countries, and controlling for participant characteristics and experiences of weight stigma, WBIS-M scores were associated with greater weight gain in the past year. Participants with higher WBIS-M scores also reported poorer mental and physical HRQOL, less eating and physical activity self-efficacy, greater engagement in eating as a coping strategy, more avoidance of going to the gym, poorer body image, and greater perceived stress. Few interaction effects were found between experiences and internalization of weight stigma. Overall, the current findings support WBI as a robust correlate of adverse weight-related health indices across six Western countries. Prospective and experimental studies are needed to determine directionality and causality in the relationship between WBI and poor health outcomes.

## Highlights

• Differences in weight bias internalization (WBI) were found between six Western countries.
• WBI was associated with weight gain in the past year across countries.
• WBI was associated with adverse weight-related health indices across countries.
• Associations with weight and health were stronger for WBI than weight stigma experiences.

## Introduction

Stigmatization of persons who are perceived to have excess weight (i.e., overweight/obesity) is prevalent across the globe (Brewis et al., 2018). Individuals with overweight/obesity are stereotyped as lazy, lacking in willpower and self-control, unattractive, and less competent than their lower weight counterparts (Puhl & Brownell, 2003). This form of bias leads to weight-based discrimination in employment, education, health care, and interpersonal relationships (Pearl, 2018). The persistent anticipation or experience of stigmatization due to weight is stressful and has implications for physiological and behavioral responses (Tomiyama, 2014). For example, experiencing weight stigma is linked to dysregulated cortisol and other markers of inflammation, which have both direct effects on mental and physical health, as well as indirect effects through changes in appetite that facilitate unhealthy eating behaviors (Tomiyama, 2014). Cumulative evidence shows that, at the population level, experiencing weight stigma is a risk factor for impaired psychological well-being, markers of chronic stress, reduced engagement in health-promoting behaviors, and weight gain over time (Pearl, 2018).

Due to the pervasive societal scorn towards persons with a higher weight, some individuals internalize negative stereotypes and devalue themselves because of their weight, known as weight bias internalization (WBI) or weight self-stigma (Durso & Latner, 2008). In comparison to experiences of weight stigma enacted by others (e.g., discrimination), less is known about the prevalence and health correlates of internalization. WBI can affect individuals across the weight spectrum and is associated with impaired mental health, including depression, anxiety, and disordered eating (Pearl & Puhl, 2018). It has also been linked to poor physical health outcomes (Pearl & Puhl, 2018), via pathways such as increased depression (Pearl et al., 2014). In addition, WBI is hypothesized to disrupt engagement in health-promoting behaviors by undermining self-efficacy to pursue and achieve goals through the self-application of negative weight-related stereotypes (e.g., laziness and lack of willpower) (Corrigan et al., 2006; Pearl & Puhl, 2018). However, studies on WBI have predominantly collected data from relatively small and restricted samples, limiting the ability to detect small effects and to generalize findings to the broader population (Pearl & Puhl, 2018).

For example, despite growing interest in WBI worldwide (Hilbert et al., 2014a; Innamorati et al., 2017; Jung et al., 2017; Lin & Lee, 2017; Meadows & Higgs, 2019; O’Brien et al., 2016; Palmeira et al., 2016), the majority of data on this topic come from the US (Pearl & Puhl, 2018). Studies of WBI conducted in different countries have varied in measures used to assess WBI and in sample characteristics (e.g., clinical versus community), making it difficult to draw conclusions about whether WBI may vary systematically across nations. Very few studies have directly compared weight stigma between different countries (Puhl et al., 2015), and none have made cross-country comparisons of WBI. As a result, knowledge of the extent to which individuals across the globe internalize weight bias, and of the health indices associated with internalization, is lacking. This is a notable gap in research as efforts to promote the public health importance of reducing weight stigma around the world continue to grow (Brewis et al., 2018).

Further, while several population-based cohort studies have included measures of weight discrimination (Jackson & Steptoe, 2017; Sutin et al., 2015), very few large-scale studies have included measures of WBI (Hilbert et al., 2014a; Puhl et al., 2018). Sample sizes have been particularly small in most investigations of WBI in clinical populations (Pearl & Puhl, 2018), with the exception of one prior US study of over 18,000 adults enrolled in a commercial weight management program (Pearl et al., 2019a, Pearl, Puhl, Himmelstein, Pinto, & Foster, 2020). Individuals who seek treatment for weight management report greater psychological distress due to weight and may be vulnerable to WBI (Friedman & Brownell, 1995; Pearl et al., 2019a). These individuals also represent a group that may be most reachable for interventions designed to reduce WBI, since they have already made contact with a treatment provider or program. However, small sample sizes in clinical studies of WBI across the world limit broad-scale investigation, and cross-country comparisons, of its associations with health and behavioral outcomes (particularly those that may be relevant to weight management, such as eating behavior, physical activity, and self-efficacy). Thus, large-scale studies of WBI in treatment-seeking adults, within and outside of the US, are needed to advance knowledge of how it may affect their weight management efforts and overall health and well-being, as well as to identify potential targets for stigma-reduction interventions.

The current study aimed to contribute knowledge of the global prevalence and correlates of WBI in treatment-seeking individuals by conducting a survey of weight stigma among members of an internationally-available weight management program. This study assessed WBI in six different Western countries (all of which culturally value lower body weight), in order to identify variation by country and determine whether health correlates of WBI remained robust across multiple nations.

## Materials and methods

### Participants

Adults enrolled in WW (formerly Weight Watchers) were simultaneously recruited from six countries: Australia, Canada, France, Germany, the United Kingdom (UK), and the United States (US). These countries were selected for their sufficiently large WW memberships to allow for recruitment of a minimum of 1000 participants in each country. WW is an empirically-validated behavioral weight management program that encourages healthy habits related to food, activity, and mindset. The study was open to WW members who were 18 years or older and had been members for at least three months.

### Procedures

Recruited participants in each country completed the same online, anonymous survey hosted by the survey site Qualtrics (Provo, UT). Surveys administered in the US, UK, Canada, and Australia were in English and required participants to be English-speaking. For participants in France and Germany, the survey was translated (and back translated) into French and German, respectively, by a professional translation services company (Language Scientific, Medford, MA). Prior to data collection for the study, all surveys were pilot-tested with small samples (<160) in each of the six countries in March 2020 to test item comprehension across languages. Data collection occurred from May to July 2020. A random set of 4000–33,000 (*M* = 23,474) members in each country were emailed each week and invited to complete the survey, which was advertised as a “survey to learn more about people's experiences related to body weight and health, including social experiences and challenges.” The protocol of this study was approved by the institutional review board at the University of Connecticut.

### Measures

**Internalization and experiences of weight stigma.** The 10-item Modified Weight Bias Internalization Scale (WBIS-M) was used to measure WBI (Durso & Latner, 2008; Pearl & Puhl, 2014). Selection of the 10-item version was based on prior psychometric data that support removal of the first item of the original 11-item scale (Hilbert et al., 2014a; Lee & Dedrick, 2016; Roberto et al., 2012). Scale items are rated from 1 (strongly disagree) to 7 (strongly agree) to assess participant endorsement of negative self-statements due to weight, reflecting both self-application of negative stereotypes (e.g., “I am less attractive than most other people because of my weight”) and lower self-worth (e.g., “I hate myself for my weight”). Scores are averaged, with higher scores indicating greater internalization. The WBIS-M is widely used in community and clinical samples (Pearl & Puhl, 2018) and has strong psychometric properties, including in the current sample (Cronbach's α values across countries ranged from 0.91 to 0.93). To assess experiences of weight stigma, participants responded to three yes/no items asking whether they had ever been discriminated against, teased or bullied, or treated unfairly because of their weight (Puhl et al., 2011). Participants who endorsed any of these items were coded as having ever experienced weight stigma, while those who endorsed no items were categorized as having never experienced weight stigma. These items and categorizations are commonly used in studies of weight stigma, including among weight management participants (Pearl, Puhl, Himmelstein, Pinto, & Foster, 2020).

**Percent weight change.** Participants reported their current weight and their weight one year ago. Percent weight change was calculated by subtracting these two values and dividing by the reported weight one year ago. This measure was included to provide a snapshot of participants’ weight trajectory, regardless of how long they had been WW members. Outliers were excluded using the 1.5 interquartile range method. Positive values signify weight gain in the past year, and negative values signify weight loss.

**Health-related quality of life (HRQOL), health behaviors, and psychosocial well-being.** The Short Form Health Survey-12 (SF-12) was used to assess *mental and physical HRQOL* (Ware et al., 1996). Responses to 12 items are divided into a mental health and physical health component summary score, respectively. Scores are transformed to a 0–100 scale based on population norms (a score of 50 represents the population mean), and higher values indicate better HRQOL.

*Eating self-efficacy* was assessed with the Weight Efficacy Lifestyle Questionnaire-Short Form (Ames et al., 2012). Participants rated their confidence (0-10) in their ability to overcome challenges to resist overeating, and higher summed scores indicate greater self-efficacy (α values across countries = 0.85–0.89). The Coping subscale of the Motivations to Eat Scale measured use of *eating to cope* with life stress (Jackson et al., 2003). Five items were rated from 1 (almost never or never) to 5 (almost always or always), with higher average scores indicating greater use of eating to cope (α values = 0.89–0.92).

The International Physical Activity Questionnaire–Short Form (IPAQ-SF) was used to assess *physical activity* (Craig et al., 2003). Across nine items, participants reported the duration (hours and minutes per day) and frequency (days per week) in the past week of vigorous- (e.g., aerobics, fast bicycling) and moderate-level activity (e.g., doubles tennis, carrying light loads), as well as walking. Total minutes engaging in each type of physical activity were computed, and outliers for each intensity level of physical activity were excluded following the IPAQ-SF scoring manual. *Exercise self-efficacy and avoidance* were measured with the Social Exercise Self-Efficacy and Gym Avoidance subscales of the Social Exercise and Anxiety Measure (Levinson et al., 2013). The self-efficacy subscale includes five items (e.g., “I am confident that I could work out/exercise with a group of people that I do not know”) rated on a 0 (not at all) to 100 (completely confident) scale, which are summed such that higher scores indicate greater exercise self-efficacy (α values = 0.83–0.93). The gym avoidance subscale includes four items (e.g., “When I go to the gym I think people are judging me”) rated on a 1 (not like me at all) to 7 (completely like me), summed, with higher values reflecting greater gym avoidance (α values = 0.87–0.91).

The 7-item Self-Monitoring subscale of the Weight Control Strategies Scale was used to assess how often participants engaged in *self-monitoring of weight, food intake, and physical activity* during the past month (Pinto et al., 2013). Items are rated on a 5-point scale (0 = *never* to 4 = *always*), and averaged, with higher values indicating more frequent self-monitoring (α values = 0.73–0.78).

*Body image* was assessed using the 7-item Appearance Evaluation subscale of the Multidimensional Body-Self Relations Questionnaire (Cash, 2000). Items are rated on a 1–5 scale and averaged, such that higher scores indicate more positive body image (α values = 0.84–0.87).

A brief version of the Perceived Stress Scale was used to assess participants’ *general life stress* in the past month (Cohen et al., 1983). The four items are rated from 1 (never) to 5 (very often) and averaged, with higher values reflecting greater perceived stress (α values = 0.77–0.82).

**Participant characteristics.** Participants reported their sex, race/ethnicity, highest level of education, relationship/marital status (including whether or not they currently had a significant other), and sexual orientation. By law, information about race/ethnicity and sexual orientation could not be collected in France or Germany. Participants reported their current height and weight, from which body mass index (BMI) was computed; implausible values were excluded. Participants also reported their subjective weight status, age of overweight onset, and their current weight management goal (lose weight versus stay the same weight). Participants reported the duration of their WW membership and their membership type: Digital (i.e., access to the WW app and online tools only); Workshop + Digital (access to WW coach-led meetings and app and online tools); or Personal Coaching + Digital (individual support from a WW coach and access to app and online tools).

### Statistical analyses

All continuous variables were checked for assumptions of normality and transformed as needed. To adjust for the large number of comparisons tested in the analyses, the significance cutoff was set at *p* ≤ 0.001. Analyses of variance (ANOVAs) and Chi-squared tests were used to identify differences in participant characteristics across countries. Correlations and ANOVAs were used to identify demographic correlates of WBIS-M scores. Descriptive statistics were calculated for WBIS-M scores across countries, and ANOVAs and subsequent pairwise comparisons were used to test for between-country differences in these scores, with and without including participant characteristics as covariates. Additional ANOVAs tested for differences in WBIS-M scores by weight status across countries. Linear regression was used to identify associations of WBI, weight stigma experiences, and their interaction with percent weight change in the past year and all other health variables, including all participant characteristics as covariates (due to their possible associations with WBI or health variables). Race/ethnicity and sexual orientation were not included as covariates in the main analyses due to aforementioned missing data in France and Germany but were included in sensitivity analyses for the US, Australia, Canada, and the UK. Analyses were conducted separately for each country in order to compare the significance and size of associations. Significant interaction terms were probed with simple effects analyses. All continuous variables in regression models were centered at the group means.

## Results

### Participants

A total of 23,415 individuals entered the survey website (Australia = 2119, Canada = 3968, France = 4656, Germany = 4149, UK = 4631, US = 3892), with the following response rates by country: US 4.9%; Australia 3.8%; Canada 5.3%; France 5.9%, Germany 4.4%, UK 4.2%. Of those, 8.0% of respondents were ineligible for the study because they declined to consent, were members of WW for less than three months, were under the age of 18, did not indicate current WW membership, or did not complete the eligibility questions. An additional 2.8% who did not report their country of residence, or reported a country different from the six included countries, were excluded.

Of the 20,871 participants who attempted to complete the survey, 6875 (32.9%) individuals were excluded for completing less than 50% of the survey or for missing key study variables, such as demographic information and questionnaires pertaining to weight stigma. After all exclusions, the final sample consisted of 13,996 adults across the six countries. Table 1 presents sample sizes per country and participant characteristics. Across countries, over 90% of participants were female and white, with an average age ranging from 47 to 57 years. Approximately 80% or more of participants’ BMIs fell within the overweight or obesity categories, with average BMIs ranging across countries from 29 to 31 kg/m^2^. Most participants identified as heterosexual and had a significant other, and participants with and without college degrees were comparably represented and aligned with population level educational attainment (OECD, 2020). Differences in participant characteristics across countries were minimal, although some did emerge for variables such as participant age, education, and duration of WW membership (Table 1).

**Table 1:** Participant characteristics by country.

	United States (*n* = 2615)		Australia (*n* = 1245)		Canada (*n* = 2708)		France (*n* = 2510)		Germany (*n* = 2613)		United Kingdom (*n* = 2305)		*F*
	*M*	*SD*	*M*	*SD*	*M*	*SD*	*M*	*SD*	*M*	*SD*	*M*	*SD*	
Age (years)	56.87	12.86	54.39	11.42	56.27	12.47	48.95	12.70	47.29	10.74	50.29	12.42	*p* < 0.001
BMI (kg/m^2^)	30.82	7.10	31.07	6.71	30.70	7.00	29.32	5.55	30.58	6.24	30.86	7.26	*p* < 0.001
Age of overweight onset	24.00	13.70	26.77	13.34	24.58	13.32	24.71	13.03	23.38	11.74	25.14	12.54	*p* < 0.001
WW membership length (years)	3.75	6.52	2.64	5.11	3.63	6.87	1.34	2.56	2.20	3.21	3.01	5.00	*p* < 0.001
	*N*	*%*	*N*	*%*	*N*	*%*	*N*	*%*	*N*	*%*	*N*	*%*	*χ*^*2*^
Sex													*p* < 0.001
Male	137	5.3	32	2.6	168	6.2	89	3.5	129	4.9	138	6.0	
Female	2472	94.5	1213	97.4	2538	93.7	2419	96.4	2483	95.0	2163	93.8	
Other	6	0.2	0	0.0	2	0.1	2	0.1	1	0.1	4	0.2	
Race													*p* < 0.001
White	2370	90.8	1209	97.2	2580	95.3	--	--	--	--	2216	96.2	
Non-White	239	9.2	35	2.8	126	4.7	--	--	--	--	87	3.8	
Education													*p* < 0.001
College Degree	1823	69.7	591	47.5	1121	41.4	1654	65.9	548	21.0	1125	48.8	
No College Degree	792	30.3	654	52.5	1587	58.6	856	34.1	2065	79.0	1180	51.2	
Current Significant Other													*p* < 0.001
Yes	2070	79.2	979	78.6	2151	79.4	1999	79.6	2167	82.9	1909	82.8	
No	537	20.5	258	20.7	550	20.3	503	20.0	442	16.9	391	17.0	
Marital Status													*p* < 0.001
Married	1836	70.2	851	68.5	1920	71.1	1358	54.1	1637	62.8	1510	65.6	
Not Married	779	29.8	391	31.5	782	28.9	1151	45.9	968	37.2	792	34.4	
Sexual Orientation													*p* = 0.175
Heterosexual	2515	96.5	1211	97.6	2603	96.4	--	--	--	--	2216	96.5	
Homosexual	46	1.8	13	1.0	52	1.9	--	--	--	--	31	1.3	
Bisexual	31	1.2	12	1.0	35	1.3	--	--	--	--	41	1.8	
Other	15	0.6	5	0.4	9	0.3	--	--	--	--	9	0.4	
BMI Category (kg/m^2^)													*p* < 0.001
<18.5	6	0.2	0	0.0	10	0.4	3	0.1	1	0.0	3	0.1	
18.5–24.9	547	20.9	194	15.6	556	20.5	516	20.6	461	17.6	457	19.8	
25–29.9	850	32.5	457	36.7	902	33.3	1031	41.1	942	36.1	794	34.4	
≥30	1212	46.4	594	47.7	1240	45.8	960	38.2	1209	46.3	1051	45.6	
Subjective Weight Status													*p* < 0.001
Very Underweight	13	0.5	6	0.5	8	0.3	6	0.2	14	0.5	9	0.4	
Underweight	6	0.2	3	0.2	8	0.3	8	0.3	8	0.3	5	0.2	
About the Right Weight	606	23.2	233	18.7	650	24.0	277	11.0	385	14.7	330	14.3	
Overweight	1394	53.3	674	54.1	1430	52.8	1564	62.3	1551	59.4	1310	56.8	
Very Overweight	583	22.3	326	26.2	596	22.0	648	25.8	649	24.8	634	27.5	
WW Membership Type													*p* < 0.001
Digital	838	32.0	585	47.0	951	35.1	1088	43.3	1605	61.4	821	35.6	
Workshop + Digital	1760	67.3	605	48.6	1739	64.2	1421	56.6	986	37.7	1448	62.8	
Personal Coaching + Digital	17	0.7	55	4.4	18	0.7	1	0.0	22	0.8	36	1.6	
Weight Goal													*p* < 0.001
Lose Weight	2253	86.2	1117	89.7	2285	84.4	2284	91.0	2346	89.8	2076	90.0	
Stay the Same Weight	342	13.1	122	9.8	398	14.7	216	8.6	251	9.6	206	8.9	
Ever Experienced Weight Stigma													
Yes	1558	59.6	698	56.1	1660	61.3	1396	55.6	1452	55.6	1336	58.0	*p* < 0.001
No	1057	40.4	547	43.9	1048	38.7	1114	44.4	1161	44.4	969	42.0	

Note. Collection of race and sexual orientation data was prohibited in France and Germany. “Not married” category includes individuals who reported being divorced, widowed, separated, never married, single (France), or in cohabitation (France). College education includes equivalent degrees across countries.

### Internalization across countries

Table 2 presents the participant demographic characteristics correlated with WBIS-M scores in the total combined sample. Higher internalization was associated with higher BMI, younger age, and younger age of overweight onset. Women and participants without a significant other reported higher internalization than men and those with a significant other. In a separate comparison, participants who were married had lower WBIS-M scores than those who were not currently married. No significant differences in WBIS-M scores were found based on race/ethnicity, education, or sexual orientation. Participants who perceived themselves to be “just about the right weight” had the lowest WBIS-M scores, followed by those who perceived themselves as underweight, then overweight and very overweight. WBIS-M scores were higher in participants who reported that they were currently trying to lose weight versus trying to maintain their weight. Participants who had been WW members for a longer duration of time, and those with Workshop + Digital memberships, had lower WBIS-M scores than those who had been in the WW program for less time or who had Digital or Personal Coaching + Digital memberships.

**Table 2:** Associations of participant characteristics with Modified Weight Bias Internalization Scale (WBIS-M) scores in total sample across all countries.

Correlations				
Variable	BMI	Age	Age of overweight onset	Duration of WW membership
WBIS-M	.40**	-.25**	-.24**	-.10**
Analyses of Variance				
Variable	Mean (SD)	*F*	*η*^*2*^_*p*_	*p*
Sex		35.86	.01	<.001
Male	3.85 (1.45)			
Female	4.32 (1.46)			
Other	3.76 (1.24)			
Race/Ethnicity^a^		2.51	.00	.11
White	4.31 (1.49)			
Not White	4.19 (1.58)			
Education		3.41	.00	.07
College degree or equivalent	4.27 (1.45)			
No college degree	4.32 (1.47)			
Current Significant Other		96.02	.01	<.001
Yes	4.24 (1.45)			
No	4.54 (1.48)			
Marital Status		144.13	.10	<0.001
Married	4.19 (1.45)			
Not Married	4.50 (1.46)			
Sexual Orientation^a^		3.68	.00	.01
Heterosexual or straight	4.30 (1.50)			
Gay, lesbian, or homosexual	4.22 (1.46)			
Bisexual	4.74 (1.60)			
Other	4.16 (1.70)			
Body Mass Index Category		852.31	.16	<.001
<18.5 kg/m^2^	3.32 (1.49)			
18.5–24.9 kg/m^2^	3.40 (1.37)			
25–29.9 kg/m^2^	4.06 (1.34)			
≥30 kg/m^2^	4.88 (1.33)			
Subjective Weight Status		1134.71	.25	<.001
Very underweight	5.36 (1.39)			
Underweight	3.46 (1.48)			
Just about the right weight	3.06 (1.27)			
Overweight	4.35 (1.28)			
Very overweight	5.30 (1.22)			
WW Membership		24.63	.00	<.001
Digital	4.40 (1.43)			
Workshop + Digital	4.22 (1.48)			
Personal Coaching + Digital	4.38 (1.52)			
Weight Goal		1398.58	.09	<.001
Lose weight	4.45 (1.40)			
Stay the same weight	3.04 (1.36)			
Ever Experienced Weight Stigma		1788.43	.11	<.001
Yes	4.72 (1.42)			
No	3.72 (1.31)			

Note: Each variable was tested in a separate correlation or analysis of variance. Logarithmic transformation was used for the variables of BMI and duration of WW membership. SD = Standard Deviation. ^a^Data not available for France or Germany. **p ≤ 0.001.

**Fig. 1:**
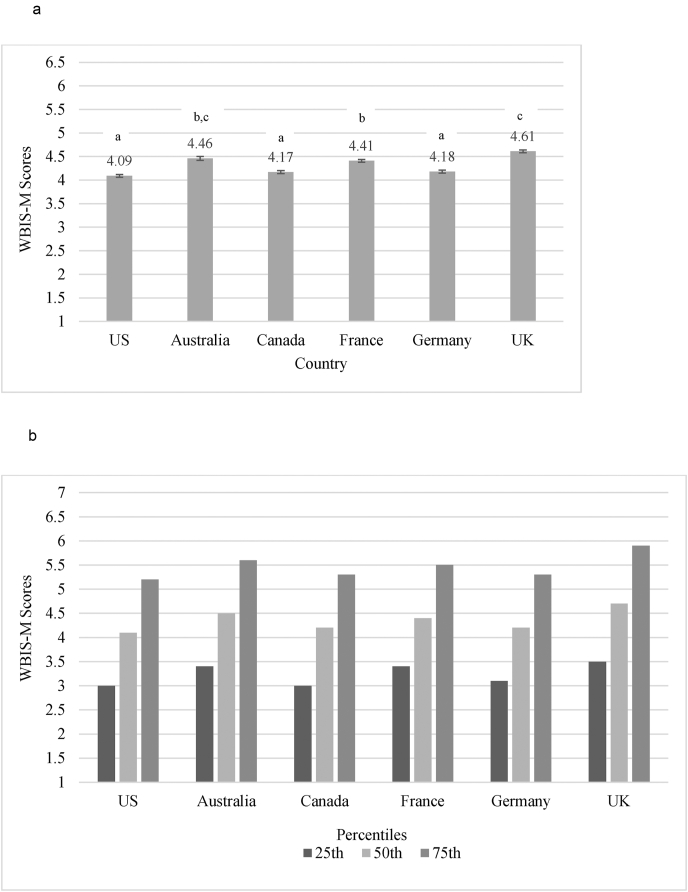
Weight Bias Internalization Across Countries. a. Unadjusted mean Modified Weight Bias Internalization Scale (WBIS-M) scores (and standard error) by country. Superscript letters indicate which means were significantly different at *p* ≤ 0.001 (countries that did not differ from one another have matching letters). When adjusting for all covariates, means (±standard error) were as follows: US = 4.19 ± 0.03; Australia = 4.45 ± 0.04; Canada = 4.23 ± 0.02; France = 4.42 ± 0.03; Germany = 4.07 ± 0.03; UK = 4.58 ± 0.03. b. Quartiles of Modified Weight Bias Internalization Scale (WBIS-M) scores by country.

**Fig. 2:**
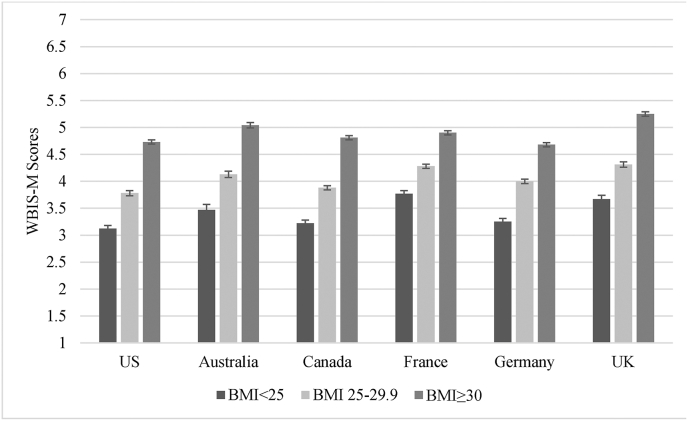
Unadjusted mean Modified Weight Bias Internalization Scale (WBIS-M) scores (and standard error) by country and BMI category. Note. Statistics for overall between-country differences in WBIS-M scores by BMI category: BMI<25: *F*(5, 2744) = 19.63, *p* < 0.001, *η*^*2*^_*p*_ = 0.04; BMI 25–29.9: *F*(5,4967) = 22.91, *p* < 0.001, *η*^*2*^_*p*_ = 0.02; BMI ≥30: *F*(5,6259) = 28.13, *p* < 0.001, *η*^*2*^_*p*_ = 0.02.

Fig. 1 presents the mean WBIS-M scores by country, as well as the scores divided into quartiles. Small but significant between-country differences in WBIS-M scores were found: *F*(5, 13,982) = 47.12, *p* < 0.001, *η*^*2*^_*p*_ = 0.02. Scores in the UK were highest, though they did not significantly differ from those in Australia. Scores in Australia were also comparable to those in France. Scores in the US, Canada, and Germany were significantly lower (*p* ≤ 0.001) than the other three countries and did not differ from one another. Fig. 2 displays the breakdown of WBIS-M scores by BMI. In all countries, WBIS-M scores were highest in participants with BMIs ≥30, followed by those with BMIs between 25 and 29.9, and lowest in participants with BMIs <25 (all *p* values < 0.001). The pattern of between-country differences in WBIS-M was largely consistent when assessed within participants belonging to each BMI category (Fig. 2).

When controlling for participant characteristics (including demographics, WW membership characteristics, BMI, age of overweight onset, and weight-stigmatizing experiences), overall differences in WBIS-M scores by country remained significant with a similar pattern of results: *F*(5,13,771) = 53.29, *p* < 0.001, *η*^*2*^_*p*_ = 0.02, total adjusted *R*^*2*^ = 0.28. Mean scores in Germany were lowest, followed by scores in the US and Canada, followed by France, Australia, and the UK (adjusted values provided in Fig. 1 footnote).

### Associations of internalization with percent weight change

Across countries, average percent weight change ranged from −3.3% to −5.2%. Small but significant differences between countries emerged, with greater weight loss in France and significantly less weight loss in the UK compared to most other countries: *F*(5,13,660) = 10.82, *p* < 0.001, *η*^*2*^_*p*_ = 0.004. Table 3 presents the linear regression results testing the associations of internalization, weight stigma experiences, and their interaction with percent weight change in the past year, controlling for all covariates (including WW membership duration). In all countries, higher WBIS-M scores were associated with greater percent weight gain in the past year. Specifically, every 1 point increase on the WBIS-M was associated with a 0.8–1.1% weight gain. The *R*^2^ contribution of WBIS-M scores (over and above that of weight stigma experiences and other covariates) ranged from 1 to 2% across countries (*p* values < 0.001). Total adjusted *R*^2^ values for the full models ranged from 10 to 15%.

**Table 3:** Linear regression analyses testing associations between weight bias internalization, weight-stigmatizing experiences, and their interaction with percent weight change in the past year, by country.

Variable	US (*n* = 2520)			Australia (*n* = 1205)			Canada (*n* = 2619)			France (*n* = 2430)			Germany (*n* = 2495)			UK (*n* = 2200)		
	B	SE	*β*	B	SE	*β*	B	SE	*β*	B	SE	*β*	B	SE	*β*	B	SE	*β*
Internalization	**.77**	**.23**	.12**	**1.12**	**.21**	.17**	**1.03**	**.22**	.16**	**1.12**	**.22**	.16**	**.97**	**.22**	.14**	**1.14**	**.23**	.17**
Experiences	-.80	.44	-.04	-1.64	.62	-.08*	-**1.41**	**.42**	-.07**	-**1.83**	**.42**	-.10**	-.66	.44	-.03	**-1.92**	**.47**	-.10**
Intern x Exper	-.23	.27	-.03	-.37	.40	-.04	-.39	.26	-.05	-.36	.28	-.04	-.45	.28	-.05	-.12	.29	-.01

Note. Intern x Exper = Internalization × Experiences interaction term. Covariates were age, sex, BMI, education (college vs. no college degree), significant other (vs. no significant other), age of overweight onset, WW membership type (ref: Digital), and WW membership duration. Significant findings are in bold. *p < 0.01 **p ≤ 0.001.

In Canada, France, and the UK, participants who reported experiencing weight stigma had approximately 1.4–1.9% greater weight loss than those who did not report such experiences. This variable was not significantly associated with percent weight change in other countries, although the direction of associations was consistent across all countries. The interaction between internalization and experiences was not significant for any country. Sensitivity analyses that included race/ethnicity and sexual orientation in the US, Australia, Canada, and UK models found the same pattern of results.

### Associations of internalization with weight-related health

Table 4 presents the results from all linear regression models examining health variables across countries. Overall, greater WBIS-M scores were associated with adverse health indices in all countries. Effect sizes were comparable across countries, with France showing somewhat weaker effects for some variables. The strongest effects across countries were found for body image (all *β* values 0.6 or greater). WBIS-M scores contributed approximately 30% of the variance in body image, and the full models accounted for 50–57% of the variance. WBIS-M scores also explained almost 20% of the variance for eating to cope in most countries (except France). Other variables for which WBIS-M scores explained at least 10% of the variance were the SF-12 mental health component summary score, eating self-efficacy, gym avoidance, and perceived stress. WBIS-M scores were associated with reduced minutes of vigorous activity in Canada and with reduced moderate activity in the UK, with very small effect sizes. No other associations between internalization and physical activity were significant. Associations between WBIS-M scores and the physical health component summary score of the SF-12 were weaker than for psychological and behavioral variables, with Germany showing the strongest effect (*β* = −0.21). Self-monitoring was also not significantly associated with WBIS-M scores in Australia or France, and across countries, the associations with self-monitoring were weaker than for most other variables. Results did not change when race/ethnicity and sexual orientation were included in the analyses for the US, Australia, Canada, and the UK.

**Table 4:** Standardized beta coefficients and *R*^*2*^ values from linear regression analyses testing associations between weight bias internalization and health variables, by country.

Variable	US			Australia			Canada			France			Germany			UK		
	*β*	ΔR^2^	Total R^2^	*β*	ΔR^2^	Total R^2^	*β*	ΔR^2^	Total R^2^	*β*	ΔR^2^	Total R^2^	*β*	ΔR^2^	Total R^2^	*β*	ΔR^2^	Total R^2^
SF-12 PCS	-.11**	.01**	.18	-.10*	.01*	.18	-.16**	.02**	.21	-.14**	.02**	.14	-.21**	.03**	.25	-.11**	.01**	.17
SF-12 MCS	-.40**	.11**	.25	-.39**	.11**	.25	-.41**	.13**	.25	-.37**	.11**	.18	-.42**	.13**	.22	-.44**	.14**	.26
Eat self-eff	-.41**	.12**	.22	-.39**	.11**	.21	-.42**	.13**	.22	-.26**	.05**	.10	-.35**	.09**	.16	-.43**	.13**	.20
Eat to cope	.53**	.19**	.35	.52**	.19**	.36	.51**	.19**	.35	.37**	.11**	.22	.49**	.18**	.35	.52**	.19**	.38
IPAQ-Vig	-.00	.00	.02	.01	.00	.03	-.09**	.01**	.03	-.07*	.00*	.03	-.08*	.00*	.02	-.07*	.00*	.04
IPAQ-Mod	-.03	.00	.02	-.01	.00	.01	-.06	.00	.03	-.05	.00	.04	-.04	.00	.02	-.10**	.01**	.03
IPAQ-Walk	-.02	.00	.03	-.01	.00	.04	-.03	.00	.04	-.06	.00	.03	-.04	.00	.04	-.03	.00	.03
Exer self-eff	-.25**	.04**	.12	-.27**	.05**	.12	-.26**	.05**	.14	-.21**	.04**	.10	-.25**	.05**	.12	-.34**	.08**	.19
Gym avoid	.38**	.10**	.25	.36**	.09**	.22	.39**	.11**	.26	.37**	.11**	.23	.39**	.11**	.25	.42**	.13**	.28
Self-monitor	-.16**	.02**	.06	-.06	.00	.04	-.12**	.01**	.04	-.01	.00	.02	-.09**	.01**	.05	-.12**	.01**	.05
Body image	-.64**	.28**	.57	-.62**	.26**	.54	-.65**	.31**	.57	-.60**	.29**	.50	-.62**	.29**	.54	-.67**	.32**	.57
Stress	.42**	.12**	.23	.46**	.14**	.24	.46**	.16**	.27	.39**	.13**	.20	.50**	.19**	.29	.46**	.15**	.25

Note. SF-12 = Short Form Health Survey-12; PCS=Physical Component Summary Score; MCS = Mental Component Summary Score; Eat self-eff = Eating self-efficacy (measured with the Weight Efficacy Lifestyles Questionnaire-Short Form); Eat to cope = Coping subscale of the Motivations to Eat Scale; IPAQ=International Physical Activity Questionnaire-Short Form; Vig = vigorous activity; Mod = moderate activity; Walk = walking; Exer self-eff = Exercise self-efficacy (measured with the Social Exercise Self-Efficacy subscale of the Social Exercise and Anxiety Measure); Gym avoid = Gym Avoidance subscale of the Social Exercise and Anxiety Measure; Self-monitor = Self-Monitoring subscale of the Weight Control Strategies Scale; Body image = Multidimensional Body-Self Relations Questionnaire-Appearance Evaluation Subscale; Stress = Perceived Stress Scale-4. Covariates were age, sex, BMI, education (college vs. no college degree), significant other (vs. no significant other), age of overweight onset, WW membership type (ref: Digital), WW membership duration, and weight-stigmatizing experiences (yes vs. no). Δ*R*^*2*^ values represent the unique contribution of WBIS-M scores to the model variance over and above that of covariates, and total adjusted *R*^*2*^ values represent the total amount of variance explained when including all covariates. Significant *β* values are in bold. *p < 0.01 **p ≤ 0.001.

Weight stigma experiences (when controlling for WBI and all other variables) were significantly (*p* ≤ 0.001) associated with several health factors: lower SF-12 mental component summary scores in France (*β* = −0.09), Germany (*β* = −0.13), and the UK (*β* = −0.07); greater eating to cope in the US, Canada, Germany, and the UK (*β* = 0.11 in Canada and Germany and 0.08 in the US and UK); greater gym avoidance in the US (*β* = 0.12), Canada (*β* = 0.08), France (*β* = 0.10), Germany (*β* = 0.14), and the UK (*β* = 0.09); greater self-monitoring in the US (*β* = 0.08), Canada (*β* = 0.09), France (*β* = 0.11), Germany (*β* = 0.11), and the UK (*β* = 0.12); and greater perceived stress in the US (*β* = 0.07), France (*β* = 0.07), and Germany (*β* = 0.08). All effect sizes were small (and smaller in magnitude than those found for WBI).

When the internalization x experiences of stigma interaction term was added to the models, it was significant for gym avoidance in the US (*β* = 0.17), Germany (*β* = 0.12), and the UK (*β* = 0.09). Simple effects analyses showed that the association between WBIS-M and gym avoidance was somewhat stronger among participants who reported experiencing weight stigma (US *β* = 0.43, WBIS-M *R*^2^ contribution = 0.14; Germany *β* = 0.40, WBIS-M *R*^2^ = 0.14; UK *β* = 0.42, WBIS-M *R*^*2*^ = 0.14) compared to participants who had not experienced weight stigma (US *β* = 0.27, WBIS-M *R*^2^ = 0.06; Germany *β* = 0.34, WBIS-M *R*^2^ = 0.10; UK *β* = 0.38, WBIS-M *R*^2^ = 0.12). In addition, a significant interaction was found for the SF-12 mental health component score in Canada (*β* = −0.11), such that effects of WBIS-M scores were stronger for participants who reported experiencing weight stigma (*β* = −0.44, WBIS-M *R*^2^ = 0.16) compared to those who did not (*β* = −0.32, WBIS-M *R*^2^ = 0.08). No other interaction terms were significant.

## Discussion

This study is the first to assess WBI in six Western countries and compare its prevalence and health correlates. Although differences were small, participants in Germany, the US, and Canada reported the lowest WBIS-M scores, followed by France, Australia, and the UK. WBIS-M scores among US respondents were comparable to those reported in a prior large-scale study of US adults in weight management (Pearl et al., 2019a). The mean WBIS-M score among German respondents in the current study, though relatively low compared to other countries, was more than 1 point higher on the 7-point scale than the mean in a prior representative German sample of adults with overweight/obesity (Hilbert et al., 2014a). This may be due to the fact that the current sample was actively engaged in weight management and thus may have had more weight-related distress than adults who were not necessarily seeking treatment (Friedman & Brownell, 1995; Jung et al., 2017). No previous studies from Canada, France, Australia, or the UK have provided data on WBI from large-scale samples for comparison.

WBIS-M scores were highest in participants with BMIs ≥30, and between-country differences were consistent across participants within each BMI category. The pattern of WBIS-M scores by country (e.g., higher scores in the UK and France and lower scores in the US) does not correspond with prevalence rates of overweight and obesity across countries; for instance, the US has the highest adult obesity prevalence rate, followed by the UK, with the lowest prevalence in France (The GBD 2015 Obesity Collaborators, 2017). Thus, higher prevalence of higher weight status does not likely have a direct impact on the internalization of weight stigma at the country level. It is possible that medical approaches to obesity and weight-related public health messages and policies, which may differ appreciably by country, could affect cultural weight attitudes (Lyn et al., 2019; Vallgarda, 2015; Wolfenden et al., 2019). Research that compares weight-related public health messages, policies, and medical guidelines across countries, along with popular media portrayals of weight, may shed light on why participants in some countries reported small but significant differences in the level to which they had internalized negative weight attitudes.

Consistent across all countries, WBI was associated with self-reported weight gain in the past year, with a 1-point increase on the WBIS-M corresponding to approximately 1% weight gain. A prior study of US WW members also found that WBI, and not weight stigma experiences, was associated with reduced weight loss and increased weight gain in the past year (Pearl, Puhl, Himmelstein, Pinto, & Foster, 2020). These data provide an observational snapshot of weight change within the broader amount of time participants may have spent in the WW program (which was several years for many participants). The current study does not provide information about how WBI affects treatment outcomes per se, as this would require prospective research that follows participants from the start of their program enrollment. In the few, small-scale clinical prospective studies of WBI that have been conducted, the relationship between WBI and reduced weight loss has been inconsistent (Lillis et al., 2019; Pearl et al., 2019b; Wott & Carels, 2010). Notably, in the current study, WBIS-M scores only accounted for 1–2% of the variance in past year weight change. The small effect sizes may explain why this relationship has not been consistently detected in smaller studies, while also suggesting the need for further research to determine the extent to which internalized weight stigma affects changes in weight in the context of weight management. There may also be temporal differences in the magnitude of effects of WBI on weight that could not be examined in the current study. For example, the first six months of weight loss treatment may elicit robust weight loss regardless of WBI, but WBI may interfere with long-term maintenance efforts in the following year (Puhl et al., 2017). Similarly, an individual's history of past weight loss attempts could affect both the magnitude of weight loss and WBI. Large-scale prospective studies of treatment-seeking adults, and long-term clinical studies that test interventions to reduce WBI, would help to clarify these effects.

WBIS-M scores also had negative associations with almost all health variables across all countries, including mental and physical HRQOL, eating and exercise self-efficacy, eating to cope, gym avoidance, body image, and perceived stress. These findings are consistent with those reported in prior studies conducted within the US, Germany, and Australia (Hilbert et al., 2014b; Hubner et al., 2015; O’Brien et al., 2016; Pearl, Puhl, Himmelstein, Pinto, & Foster, 2020; Vartanian, Pinkus, & Smyth, 2016). The substantial variance in body image accounted for by internalization suggests potential overlap in these constructs, which requires further attention in future work (Meadows & Higgs, 2020). Weak and inconsistent associations were found for self-monitoring of weight-related behaviors and self-reported physical activity (measured with the IPAQ-SF). Self-monitoring is a core feature of the WW program, which may have contributed to the lack of variation by level of internalization. The IPAQ-SF is validated and widely used but is still subject to bias of over-reporting (Prince et al., 2008; Sallis & Saelens, 2000). Although outliers were excluded following scoring guidelines, several study participants commented on finding the measure confusing to complete, thus lowering confidence in the accuracy of the measure. Future studies that use interview self-report or objective assessments of physical activity would enhance accuracy in measurement of this health behavior for closer examination in relation to WBI.

As in some prior studies (Pearl et al., 2015, Pearl, Puhl, Himmelstein, Pinto, & Foster, 2020), WBI did not significantly interact with experiences of weight stigma for most measures. In addition, non-significant or positive associations between experiences of weight stigma and some health variables (including weight loss) were found. However, the effect sizes were very small, and the findings must be interpreted with caution in the context of other large-scale studies – both within and outside of the US – that have shown associations between weight stigma experiences (such as teasing and discrimination) and adverse health outcomes over time (including weight gain) (Hubner et al., 2016; Jackson et al., 2014; Sutin & Terracciano, 2013). Findings from the current study do not support the notion that weight stigma is associated with better health; rather, the findings suggest a more complex relationship that may be contingent on which aspects of weight stigma are measured (e.g., discrete events vs. everyday microaggressions), which other variables are included in statistical models (e.g., internalization), and whether the focus is on the acute effects of weight stigma on weight loss efforts (measured at a single point, as in the current study) versus its cumulative effects over time.

Strengths of the current study include the large sample of adults engaged in weight management, thus allowing for study of a population usually restricted to highly-controlled clinical trials with small samples and limited generalizability. The cross-country assessment of WBI was also novel and provided data on means and quartiles of WBIS-M scores in six Western countries in which lower weight is culturally valued. Identical measures were used across countries and sample characteristics were highly similar, strengthening the comparisons. The inclusion of several psychological, behavioral, and health-related measures relevant to weight management also advances knowledge of how WBI specifically relates to adults’ efforts to lose or maintain their weight.

Study limitations include the cross-sectional and observational design, reliance solely on self-report measures, and use of retrospective recall to determine changes in weight. Due to the study design, no conclusions about causality or directionality of effects can be made. For example, individuals may experience weight gain due to internalized negative beliefs about themselves due to their weight (e.g., that they are lazy and lack willpower) and lower self-worth, or they may develop these self-stigmatizing beliefs because they have gained weight. Future studies that follow adults prospectively in their weight management efforts would allow for investigation of potential mechanisms in the relationship between WBI and weight change (tested with statistical mediation of behavioral and psychological process variables measured at different time points) and could test whether these longitudinal patterns are similar across different countries. Experimental studies – particularly those that test stigma-reduction interventions – would help to determine the causal effects of WBI on weight-related health outcomes. The small percentage of WW members who responded to the survey may not be representative of all WW members, or of adults who do not participate in commercial weight management programs (although similar associations between WBI and body image, self-efficacy, and HRQOL have also been shown in community samples) (Pearl & Puhl, 2018). Our data do not provide information about whether individuals who access commercial weight management programs have elevated levels of WBI, which would require a matched design of adults who are and are not enrolled in such programs. We also did not assess whether participants had enrolled in other weight management programs or interventions which might affect their weight or WBI. Importantly, exploration of WBI in non-Western and non-majority white countries also warrants further investigation. Finally, data collection occurred during the COVID-19 pandemic. It is not yet known how weight and health behaviors have been affected by COVID-19 on a global scale; however, the consistency of this study's findings with past reports suggest that they are generalizable beyond the pandemic.

The robust associations between WBI and health across six Western countries have important implications for clinical care and public health. Health professionals must consider the potential for their messages about weight to be internalized in a negative way, and for internalization's potential ironic effects on the very weight management behaviors and outcomes that the messages are meant to encourage. Careful attention to how public health discussions of weight are framed, such as avoiding promotion of blame or shame towards individuals with a higher weight, is needed at a global scale to prevent sociocultural messages from facilitating the internalization of weight stigma. Individual treatment providers must also be aware of the negative societal messages received by patients with obesity and be attuned to whether their patients express signs of internalized weight stigma. Offering support, encouragement, and evidence-based treatment are imperative to preventing further exacerbation of self-stigmatization due to weight.

### Conclusions

Levels of WBI varied to a small but significant degree across six Western countries, with lower scores in the US, Germany, and Canada and higher scores in France, Australia, and the UK. Across all countries, WBI was associated with greater self-reported weight gain in the past year and greater endorsement of adverse psychological, behavioral, and health-related outcomes. Prospective and experimental research is needed to determine the temporal and causal effects of WBI on weight-related health in the context of weight management.

## Data statement

Data will be made available upon request.

## Ethical statement

This study was approved by the Institutional Review Board at the University of Connecticut.

## Funding

This study was funded by a grant from 10.13039/100015519WW International, Inc. to the 10.13039/100007710University of Connecticut on behalf of RMP. RLP is supported by a K23 Mentored Patient-Oriented Research Career Development Award from the 10.13039/100000050National Heart, Lung, and Blood Institute of the 10.13039/100000002NIH (#K23HL140176).

## CRediT authorship contribution statement

**Rebecca L. Pearl:** Methodology, Formal analysis, Writing - original draft. **Rebecca M. Puhl:** Conceptualization, Funding acquisition, Investigation, Methodology, Writing - review & editing. **Leah M. Lessard:** Data curation, Project administration, Writing - review & editing. **Mary S. Himmelstein:** Methodology, Writing - review & editing. **Gary D. Foster:** Conceptualization, Methodology, Resources, Supervision, Writing - review & editing.

## Declaration of competing interest

RMP received funding for the current study from WW International, Inc. RLP has served as a consultant for and received grant funding, outside of the current study, from WW International, Inc. GDF is an employee and shareholder of WW International, Inc.
